# Human circular RNA hsa_circRNA_101705 (circTXNDC11) regulates renal cancer progression by regulating MAPK/ERK pathway

**DOI:** 10.1080/21655979.2021.1955579

**Published:** 2021-07-24

**Authors:** Chong-Yi Yang, Jie Wang, Jian-Qiu Zhang, Hong-miao Cai

**Affiliations:** a Department of Urology, Ninghai First Hospital, Zhejiang, China; b Department of Anesthesia, Shanghai East Hospital, Tongji University School of Medicine, Shanghai, China

**Keywords:** Circular RNAs, circTXNDC11, renal cancer, MAPK/ERK

## Abstract

Circular RNAs (circRNAs) play essential roles in the progression of human tumors, including renal cell carcinoma (RCC). The present study aimed to explore the functions and potential mechanisms of human circular RNA hsa_circRNA_101705 (circTXNDC11) in RCC. Quantitative real-time polymerase chain reaction (qRT-PCR) was applied to measure circTXNDC11 expression in RCC tissues and cell lines. RNase R and actinomycin D assays were conducted to analyze the characteristic of circTXNDC11. Cell Counting Kit-8 (CCK-8) assay, colony formation assay, and transwell invasion assay were performed to assess cell proliferation and invasion abilities. Western blotting was applied to assess the levels of MEK and ERK proteins in RCC cells. Murine xenograft model assay was conducted to deduce the role of circTXNDC11 in vivo. The current data showed that circTXNDC11 was overexpressed in RCC tissues and cells. The overexpression of circTXNDC11 is linked to advanced TNM stage and lymph node metastasis of renal cancer. Knocking down circTXNDC11 suppressed cell proliferation and invasion in vitro and reduced tumor growth in vivo. Mechanistically, circTXNDC11 promoted RCC growth and invasion by activating the MAPK/ERK pathway. Thus, the current findings identified circTXNDC11 as a novel regulator of RCC tumorigenesis through the regulation of the MAPK/ERK pathway, offering a potential therapeutic target for RCC treatment.

## Introduction

Renal cell carcinoma (RCC) is a fatal disease contributing to a high number of deaths from urinary tract-related tumors worldwide [1,2]. Although recent treatment modalities have improved the overall survival of RCC patients, the five-year overall survival rate of metastatic or advanced RCC remains unsatisfactory [3,4]. Therefore, it is crucial to explore the pathogenesis of RCC, search for new diagnostic markers, and identify useful therapeutic targets.

Circular RNAs (circRNAs), a novel class of non-coding RNAs with 3ʹ- and 5ʹ-ends covalently linked in a closed-loop structure, was found in all eukaryotic cells [5,6]. Increasing evidence has shown that circRNAs exert a tumor-suppressive or carcinogenic role in diversified malignant cancers, including RCC [7,8]. For example, Li et al. found that circPRRC2A enhanced angiogenesis and metastasis of RCC through epithelial-mesenchymal transition and upregulated TRPM3 expression [9]. Wang et al. showed that circDHX33 promoted RCC proliferation and invasion via regulating the miR-489-3p/MEK1 axis [10]. Chen et al. demonstrated that circRNA cRAPGEF5 repressed growth and metastasis of RCC by targeting the miR-27a-3p/TXNIP axis [11]. However, the roles and underlying mechanisms of circTXNDC11 in RCC are yet unclear.

In the present study, we identified a novel circular RNA hsa_circRNA_101705 (circTXNDC11) in RCC, which is upregulated and associated with advanced clinical features of RCC patients. Furthermore, additional experiments have shown that circTXNDC11 promoted RCC progression via regulating the MAPK/ERK axis. In summary, circTXNDC11 serves as an oncogene that could be a potential target for RCC treatment.

## Methods

### Clinical samples

The present study was approved by the Ethics Committee of Ninghai First Hospital and Shanghai East Hospital. Overall, 30 pairs of RCC tissues and adjacent nonmalignant tissues were extracted from RCC patients at Ninghai First Hospital and Shanghai East Hospital. None of the patients received preoperative chemotherapy and radiotherapy. All specimens were removed and immediately preserved in −196°C liquid nitrogen until used for RNA extraction. Informed consent was acquired from the patients.

### Cell culture and transfection

Human proximal tubule epithelial cell lines HK-2 and RCC (ACHN, 786-O, A498, Caki1, and Caki2) were purchased from the Chinese Academy of Sciences (Shanghai, China), cultured in Dulbecco’s modified Eagle medium (DMEM, Gibco, Carlsbad, CA, USA) supplemented with 10% fetal bovine serum (FBS, Gibco), and incubated at 37°C in an incubator containing 5% CO_2_.

Small interfering RNAs (si-RNAs) against circTXNDC11 (si-circTXNDC11#1 and si-circTXNDC11#2) and their corresponding controls (si-NC) were synthesized by GenePharma (Shanghai, China). Transfection was performed using Lipofectamine 3000 (Invitrogen, Thermo Fisher, Waltham, MA, USA), according to the manufacturer’s instructions.

### Real-time quantitative PCR

Total RNAs from RCC tissues and cells were extracted using TRIzol (Thermo Fisher), as described by the manufacturer and previous study [12]. PrimeScript RT kit (TaKaRa, Dalian, China) was used to reverse-transcribe the total RNA into cDNA, followed by RT-qPCR performed using the Power SYBR-Green Premix kit (TaKaRa), as described by the manufacturer. *GAPDH* and *U6* served as the internal controls. 2^−ΔΔCt^ method was used for quantifying the gene levels.

### Cell counting kit-8 (CCK-8) assay

The effect of circTXNDC11 on the viability of RCC cells was explored using the CCK-8 kit (Dojindo, Japan). Briefly, transfected RCC cells were seeded into a 96-well plate and grown for 24, 48, 72, and 96 h. Then, cells were incubated with CCK-8 reagent for 4 h, and the absorbance was measured at 450 nm on a microplate reader (Bio-Rad, Hercules, CA, USA).

### Cell apoptosis assay

Transfected RCC cells (2 × 10^5^ cells/well) were inoculated in 6-well plates. Subsequently, the cells were collected by centrifugation, resuspended in Annexin V binding buffer, and stained using fluorescein isothiocyanate (FITC)/propidium iodide (PI) staining kit (Ribobio, Shanghai, China) according to the manufacturer’s instructions. Cell apoptosis was analyzed using a flow cytometer (Beckman, Chaska, MN, USA).

### Transwell assay

The Matrigel (Corning, NY, USA) covered chambers (Corning, 8.0-μm-pore) were used to explore cell invasion capacity. Briefly, 1 × 10^5^ transfected RCC cells were seeded in the FBS-free medium in the upper chambers, whereas the lower compartments were filled with DMEM medium supplemented with 20% FBS. After 24 h, the cells that passed through the membranes were fixed with 4% paraformaldehyde and stained using crystal violet (Sigma–Aldrich). Finally, the number of invaded cells was counted under an inverted light microscope (Olympus, Tokyo, Japan).

### Xenograft model

Specific pathogen-free grade (SPF) BALB/c nude mice (4–6-week-old) were commercially provided by the Institute of Laboratory Animals at the Chinese Academy of Medical Sciences (Shanghai, China). To establish tumor xenografts, transfected RCC cell suspensions (2 × 10^6^/mouse) were subcutaneously inoculated into the nude mice. The tumor volume was calculated every 7 days. After 6 weeks, the mice were sacrificed to extract the tumor tissues for volume and weight analysis [13]. The study was approved by the ethics committee of Ninghai First Hospital and Shanghai East Hospital.

### Western blot

Total proteins in RCC cells were isolated using RIPA lysis buffer, separated on a 10% sodium dodecyl sulfate-polyacrylamide gel electrophoresis (SDS-PAGE), and transferred to polyvinylidene difluoride (PVDF) membranes. Then, the membranes were blocked for 2 h at room temperature using 5% nonfat milk and probed with primary antibodies at 4°C overnight in TBST, followed by incubation with horseradish peroxidase-labeled secondary antibody at 25°C for 1 h [14]. Enhanced chemiluminescence (ECL) was employed to visualize the immunoreactive protein bands.

### Statistical analysis

Data were analyzed using SPSS software V. 22.0 (IBM, Armonk, NY, USA). The data are expressed as mean ± standard deviation (SD). The differences between the two groups were evaluated using Student’s t-test, whereas analysis of variance (ANOVA) with Tukey’s post hoc test was used for multiple groups. P < 0.05 indicated a statistically significant difference.

## Results

### circTXNDC11 was overexpressed in RCC

**Figure 1.**
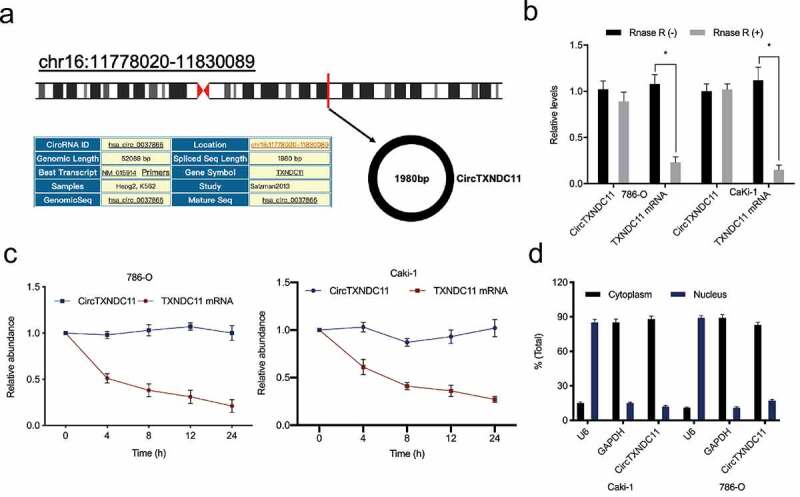
Characteristics of circTXNDC11 in RCC

Recently, Cen et al. showed that hsa_circRNA_101705 was upregulated in RCC [15]. Human circular RNA hsa_circRNA_101705 (circTXNDC11), located on chr16:11,778,020–11,830,089, is a transcript of *TXNDC11* gene (Figure 1(a)). RNase R assay results showed that compared to linear TXNDC11, circTXNDC11 is more resistant to RNase R (Figure 1(b)). Subsequently, RCC cells were treated with Actinomycin D, which revealed that circTXNDC11 has a longer half-life than liner TXNDC11 (Figure 1(c)). Cytoplasmic/nuclear separation assay showed that circTXNDC11 is almost exclusively located in the cytoplasm of RCC cells (Figure 1(d)).

(a) Schematic illustration of circTXNDC11. (b, c) qRT-PCR for the relative expression of circTXNDC11 and TXNDC11 in RCC cells treated with RNase R and Actinomycin D. (d) The cellular location of circTXNDC11 in RCC cells; *P < 0.05.

**Figure 2.**
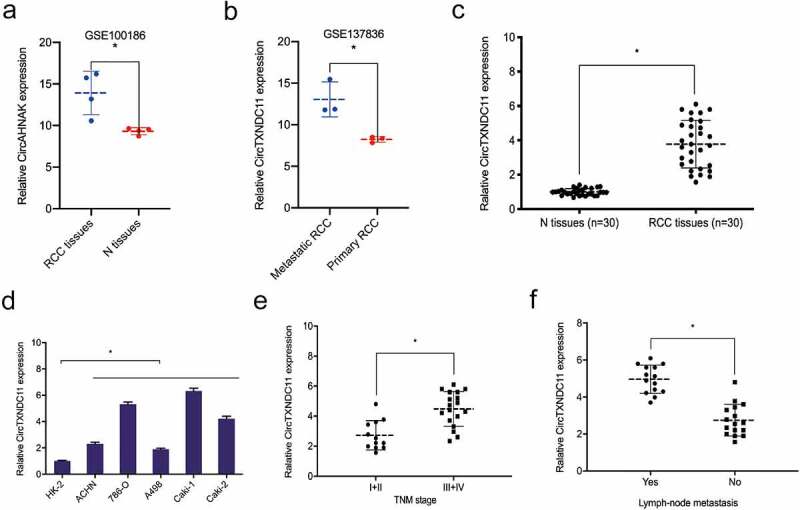
Relative expression of circTXNDC11 in RCC

Next, we explored the expression of circTXNDC11 in RCC progression. GSE100186 dataset revealed that circTXNDC11 was overexpressed in RCC tissues compared to adjacent non-tumor tissues (N) (Figure 2(a)). GSE137836 dataset showed that circTXNDC11 expression was significantly higher in metastatic than primary RCC tissues (Figure 2(b)). Furthermore, we studied the expression of circTXNDC11 in our RCC dataset. qRT-PCR showed that circTXNDC11 was overexpressed in RCC tissues and cell lines (Figure 2(c,d)). Based on correlation analysis, the overexpression of circTXNDC11 was associated with advanced TNM stage and lymph node metastasis of RCC patients (Figure 2(e,f)). These findings indicated that circTXNDC11 was stable and might play a role in RCC carcinogenesis.

(a, b) circTXNDC11 expression in GSE100186 (a) and GSE137836 (b) datasets. (C, D) qRT-PCR detected the expression of circTXNDC11 in RCC tissues (c) and cell lines (d). (e, f) High circTXNDC11 expression was associated with advanced TNM stage (e) and lymph-node metastasis (f) in RCC; *P < 0.05.

### circTXNDC11 knockdown suppressed RCC cells proliferation and invasion

**Figure 3.**
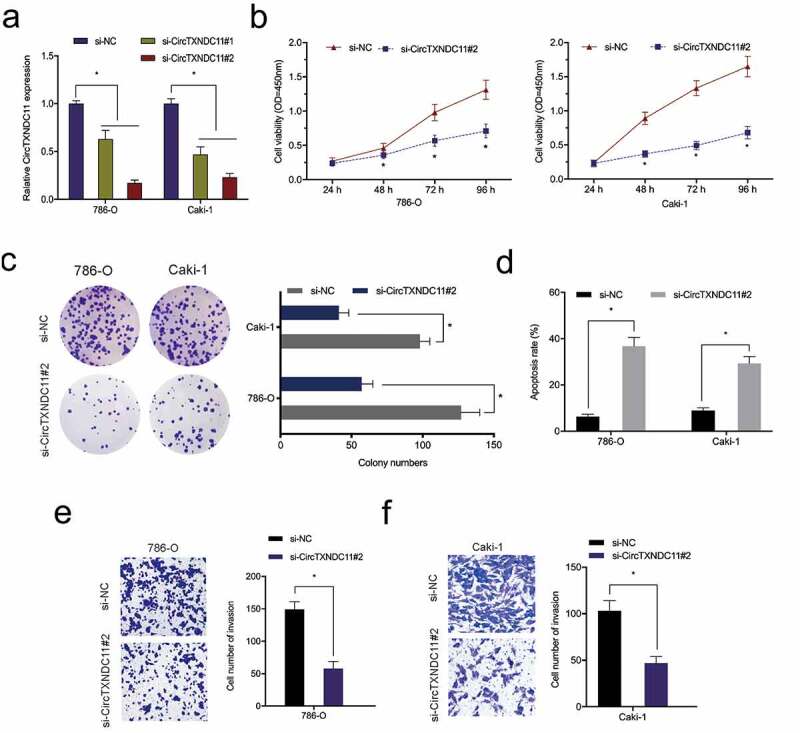
circTXNDC11 knockdown inhibited the proliferation and invasion of RCC cells in vitro

To explore the exact roles of circTXNDC11, si-circTXNDC11 was transfected into 786-O and CaKi-1 cells (Figure 3(a)). CCK-8 assay showed that circTXNDC11 suppression repressed the viability of 786-O and CaKi-1 cells (Figure 3(b)). The colony formation assay indicated that circTXNDC11 interference significantly restrained the colony formation ability of 786-O and CaKi-1 cells (Figure 3(c)). Flow cytometry revealed that the inhibition of circTXNDC11 enhanced the apoptosis of 786-O and CaKi-1 cells (Figure 3(d)). In addition, transwell assay revealed that circTXNDC11 silencing repressed the invasion ability of 786-O and CaKi-1 cells (Figure 3(e,f)). Collectively, circTXNDC11 knockdown repressed the progression of RCC cells in vitro.

(a) qRT-PCR for the transfection efficacy of si-circTXNDC11 in RCC cells. (B–F) Effect of circTXNDC11 knockdown on proliferation (b) colony formation (c), apoptosis rate (d), and metastasis (e, f) of RCC cells in vitro; *P < 0.05.

**Figure 4.**
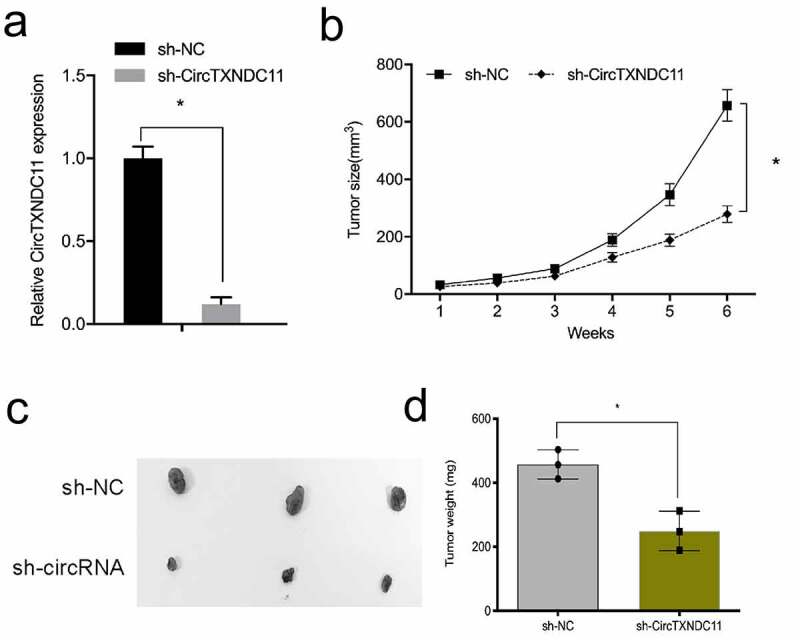
circTXNDC11 silencing reduced RCC cell growth in vivo

Next, the influence of circTXNDC11 in the in vivo tumor growth was explored. 786-O cells stably expressing sh-circTXNDC11 were subcutaneously injected in nude mice with tumor xenografts (Figure 4(a)). circTXNDC11 suppression reduced the growth of RCC cells in vivo (Figure 4(b)). In addition, circTXNDC11 knockdown reduced tumor volume and tumor weight compared to sh-NC control group (Figure 4(c,d)). These findings demonstrated that circTXNDC11 silencing blocked tumorigenesis in vivo.

(a) Transfection efficacy of sh-circTXNDC11 in 786-O cells. (b) circTXNDC11 suppression reduced the growth of RCC tumor in nude mice. (c, d) circTXNDC11 inhibition decreased the volume and weight of xenograft tumors in nude mice; *P < 0.05.

### Functional pathway analysis of circTXNDC11

**Figure 5.**
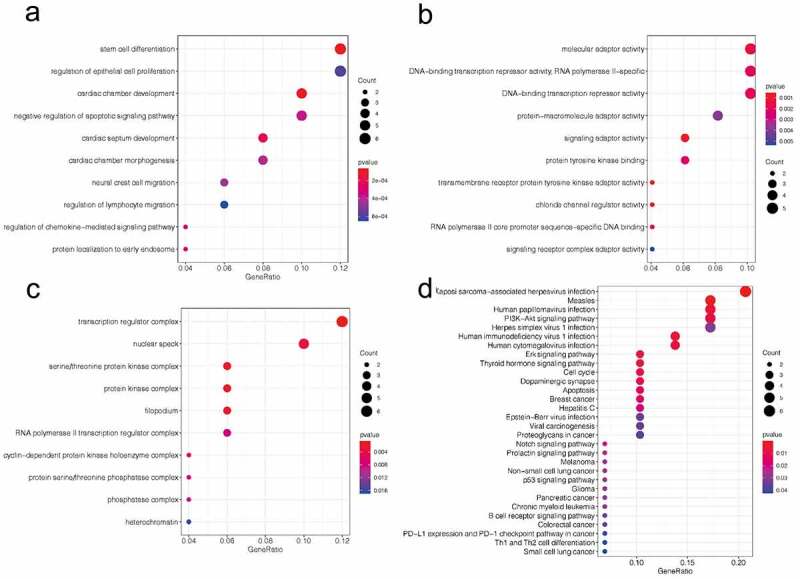
GO and KEEG pathway analysis of circTXNDC11 in RCC

Functional enrichment analysis was conducted to explore the potential function of circTXNDC11. Gene Ontology (GO) terms of biological processes (BP) were represented by terms, such as stem cell differentiation, regulation of epithelial cell proliferation, and regulation of lymphocyte migration (Figure 5(a)). The enriched molecular function (MF) terms were molecular adaptor activity, protein-macromolecule adaptor activity, and signaling adaptor activity (Figure 5(b)). The cellular components (CC) were represented by transcription regulator complex, nuclear speck, protein kinase complex, and cyclin-dependent protein kinase holoenzyme complex (Figure 5(c)). KEGG pathway showed that the target genes were enriched in the following signaling pathways: PI3K-Akt, Erk, Notch, and p53 (Figure 5(d)). These findings demonstrated that circTXNDC11 plays a critical role in tumor progression.

(a–c) The top 10 processes of circTXNDC11 enrichment in biological process (BP), molecular functions (MF), and cellular component (CC), respectively, by GO analysis. (d) The top 30 pathways of circTXNDC11 enrichment predicted by KEEG analysis.

### circTXNDC11 modulates RCC-related processes via the MAPK/ERK axis

**Figure 6.**
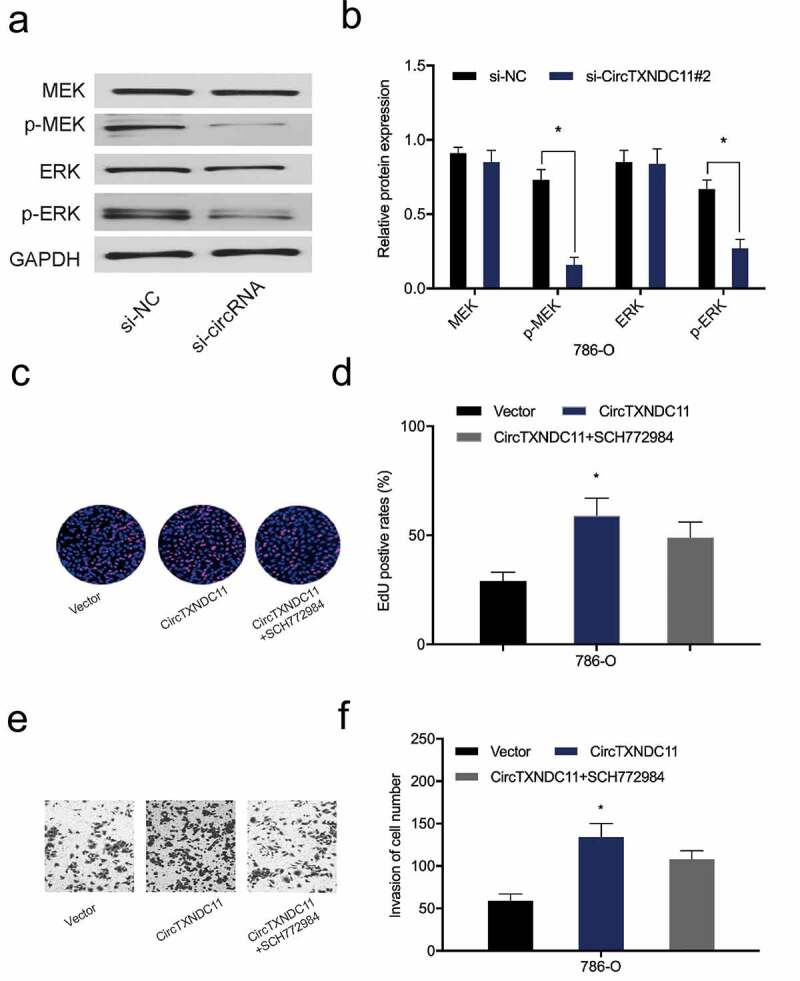
circTXNDC11 activated the MAPK/ERK pathway in RCC

Next, we examined the effect of circTXNDC11 on the MAPK/ERK signaling cascade, which is essential to the proliferation, survival, and differentiation of many cancer types, including RCC [16]. Western blot analysis revealed that circTXNDC11 inhibition repressed the expression of phospho-ERK (p-ERK) and phospho-MEK (p-MEK) proteins in 786-O cells (Figure 6(a,b)). Furthermore, functional rescue assays showed that ERK inhibitor (SCH772984) abolished the effects of circTXNDC11 overexpression on 786-O cell proliferation and invasion (Figure 6(c-f)). Overall, these findings demonstrated that circTXNDC11 promotes RCC cell progression via MAPK/ERK signaling pathway regulation.

(a, b) The effects of si-circTXNDC11 on the expression of p-ERK, ERK, p-MEK, and MEK proteins in 786-O cells. (c–f) ERK inhibitor (SCH772984) abolished the biological effects of circTXNDC11 overexpression in 786-O cell proliferation (c, d) and invasion (e, f); *P < 0.05.

## Discussion

Emerging studies have unveiled that dysregulation of circRNAs plays a critical role in tumorigenesis [17,18]. For example, Zong et al. documented that the overexpression of circRNA_102231 was associated with advanced clinical features and poor prognosis of lung cancer patients [19]. Zhang et al. reported that upregulated circRNA_069718 expression promoted growth and invasion of triple-negative breast cancer cells via the Wnt/β-catenin pathway [20]. Liu et al. reported that circSERPINA3 promoted proliferation and invasion of nasopharyngeal carcinoma cells by regulating the miR-944/MDM2 axis [21]. However, the roles and underlying mechanisms of circRNAs in tumor progression remain largely unclear.

In the present study, we identified a novel circRNA circTXNDC11. The stability of circTXNDC11 was confirmed by its stable expression under RNase R digestion and Actinomycin D treatment. Next, the dysregulated expression and biological function of circTXNDC11 were investigated. The results showed that circTXNDC11 was overexpressed in RCC tissues and cell lines, and high circTXNDC11 expression was correlated with advanced TNM stage and lymph node metastasis of RCC. Furthermore, functional assays showed that circTXNDC11 knockdown effectively inhibited cell proliferation and invasion of RCC cells in vitro and suppressed tumor growth in vivo. These data suggested that circTXNDC11 acted as a tumor oncogene in RCC progression.

Mitogen-activated protein kinases (MAPKs) are serine/threonine protein kinases consisting of ERK1/2, MEK1/2, and P38 [22,23]. Several studies have shown that the activation of the MAPK/ERK pathway promotes proliferation, survival, and migration but inhibits the apoptosis of multiple cancer cell types. For instance, Xiao et al. reported that by targeting SPRY2, miR-330-5p promoted the proliferation of liver cancer cells via the MAPK/ERK axis [24]. Zhu et al. found that lncRNA SDPR-AS inhibited cell proliferation and invasion of non-small cell lung cancer by modulating SDPR via the p38 MAPK/ERK axis [25]. Gao et al. demonstrated that hsa_circRNA_0006528/miR-7-5p promoted the progression of breast cancer cells by activating the MAPK/ERK pathway [26]. However, whether circTXNDC11 affects MAPK/ERK pathway in RCC progression is yet to be clarified.

In the present study, we first predicted the mechanism underlying circTXNDC11 in RCC using bioinformatics. The potential processes and pathways, such as epithelial cell proliferation, molecular adaptor activity, protein kinase complex, cyclin-dependent protein kinase, PI3K-Akt signaling pathway, and Erk signaling pathway, were regulated by circTXNDC11. Next, we verified the effects of circTXNDC11 on the MAPK/ERK axis in RCC cells and found that circTXNDC11 inhibition decreased the levels of phospho-ERK (p-ERK) and phospho-MEK (p-MEK) in RCC cells. SCH772984 (specific inhibitor of ERK) abolished the effects of circTXNDC11 overexpression on RCC cells proliferation and invasion. These data suggested that circTXNDC11 promotes RCC carcinogenesis by triggering the MAPK/ERK signaling cascade.

## Conclusion

Overall, the current study confirmed that circTXNDC11 was upregulated in RCC, and its suppression impeded the proliferation and metastasis of RCC. This study clarified that circTXNDC11 promoted RCC tumorigenesis via the circTXNDC11/MAPK/ERK cascade, providing a novel therapeutic target for the clinical treatment and prognosis of RCC.

## Highlights

1. CircTXNDC11 is overexpressed in RCC
2. CircTXNDC11 promotes the proliferation and invasion in RCC cells
3. CircTXNDC11 activates the MAPK/ERK pathway in RCC cells
